# Beer Compared with Other Alcoholics

**Published:** 1890-01

**Authors:** 


### Beer Compared With Other Alcoholics.

For some years a decided inclination has been apparent all over the country to give up the use of whisky and other strong alcoholics, using as a substitute beer and other compounds. This is evidently founded on the idea that beer is not harmful, and contains a large amonut of nutriment; also that bitters may have some medical quality which will neutralize the alcohol which it conceals, etc. These theories are without confirmation in the observation of physicians. The use of beer is found to produce a species of degeneration of all the organs. Profound and deceptive fatty deposits, diminished circulation, conditions of congestion, and perversion of functional activities, local inflammations of both liver and kidneys are constantly present. Intellectually a stupor amounting to almost paralysis arrests the reason, changing all the higher faculties into a mere animalism, sensual, selfish, sluggish, varied only with paroxysms of anger that are senseless and brutal. In appearance, the beer-drinker may be the picture of health, but in reality he is most incapable of resisting disease. A slight injury, a severe cold, or a shock to the body or mind, will commonly prove acute disease, ending fatally. Compared with inebriates who use different kinds of alcohol, he is more incurable, and more generally diseased. The constant use of beer every day gives the system no recuperation, but steadily lowers the vital forces. Recourse to beer as a substitute for other forms of alcohol merely increases the danger and fatality.—*Scientific American.*

